# Accuracy of Different Putty-Wash Impression Techniques with Various Spacer Thickness

**DOI:** 10.5005/jp-journals-10005-1131

**Published:** 2012-02-24

**Authors:** Anshul Chugh, Aman Arora, Vijay Pratap Singh

**Affiliations:** Assistant Professor, Department of Prosthodontics and Implantology Government Dental College, Uh-7, Medical Campus, Rohtak, Haryana India, e-mail: dr.anshulchugh@rediffmail.com; Professor and Head, Department of Prosthodontics, DAV Dental College, Yamunanagar, Haryana, India; Professor, Department of Prosthodontics, DAV Dental College Yamunanagar, Haryana, India

**Keywords:** Putty-wash techniques, Impression techniques, Wash space, Different spacers thickness, Comparison, Accuracy

## Abstract

One of the most important steps is accurate impression making for fabrication of fixed partial denture.

The two different putty-wash techniques that are commonly used are: (1) Putty-wash one-step technique, (2) putty-wash two-step technique.

A uniform wash space is needed for an accurate impression. Nissan et al recommended the use of two-step technique for accurate impression making as there is uniform wash space for the light body material to polymerize.

The aim of the present study was to compare the accuracy of stone casts obtained from different putty-wash impression techniques using various spacer thickness.

The critical factor that influences the accuracy of putty-wash impression techniques is the controlled wash bulk which is absent in one-step putty-wash impression technique and with polyethylene spacer was used.

**How to cite this article:** Chugh A, Arora A, Singh VP. Accuracy of Different Putty-Wash Impression Techniques with Various Spacer Thickness. Int J Clin Pediatr Dent 2012;5(1):33-38.

## INTRODUCTION

One of the most important steps is accurate impression making for fabrication of fixed partial denture. The two different putty-wash techniques that are commonly used are: (1) putty-wash one-step technique, (2) putty-wash two- step technique. A uniform wash space is needed for an accurate impression. Nissan et al recommended the use of two-step technique for accurate impression making as there is uniform wash space for the light body material to polymerize.

Putty acts as a tray for wash material. Light body being less viscous has good flow to record the fine details resulting in an accurate impression. An accurate impression produces the stone casts with minimal dimensional change in regard to the vertical and horizontal dimension between the prepared abutments. Clinical success of fixed prosthodontic procedure is dependent upon the dimensional accuracy of elastomeric impression material and impression procedures**.**

## AIMS AND OBJECTIVES

- To compare the accuracy of various impression techniques made with putty-wash impression material.
- To determine the effect of wash space on the accuracy of impressions made with different techniques.
- Clinical recommendations based on study and observation.

## MATERIALS AND METHODS

In the present study, putty-wash impression techniques with different spacer thickness of 1 and 2 mm and polyethylene spacer has been used. The two putty-wash impression techniques that have been compared for dimensional accuracy are one-step and two-step procedures.

### Materials

1. Master model, containing three complete crown fixed partial denture abutment preparations.
2. Six metal copings, three each of 1 and 2 mm thickness.
3. Polyethylene separating sheets.
4. Perforated metal tray.
5. Addition silicone impression material. (Flextime, Heraeus Kulzer) (easy putty and light-bodied polyvinyl siloxane).
6. Tray adhesive (Heraeus Kulzer, universal adhesive).
7. Die stone (Kalrock, super hard die stone class IV, Kalabhai Karson, Mumbai).
8. Debubblizer (Dentofill).

### Armamentarium

**Fig. 1:**
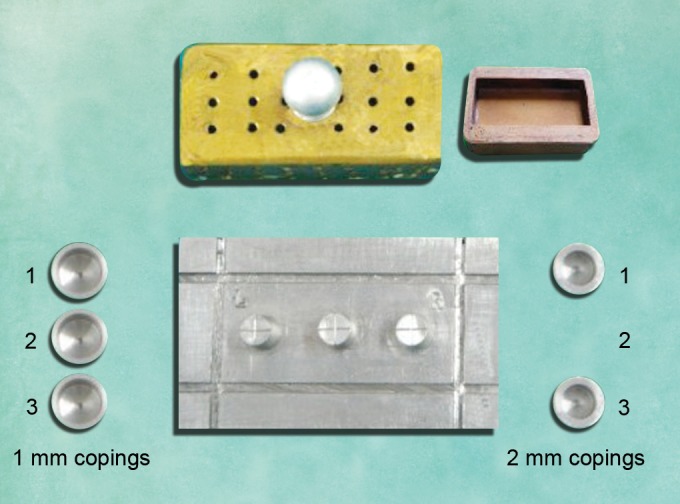
Armamentarium used

1. Vaccum mixer
2. Automatic mixing syringe and dispensing gun (Heraeus Kulzer)
3. Vibrator
4. Rubber bowl
5. Mixing spatula
6. Base former
7. Stopwatch
8. Coordinate measurement machine (CMM, Llyod, Germany) (Fig. 1).

## PREPARATION OF MASTER MODEL

A metal master model, containing three complete crown fixed partial denture abutment preparations, was fabricated for making the measurements. The abutments were prepared with occlusal taper of 6° and two perpendicular cross grooves on the occlusal surface as reference points for taking measurements.

### Grouping of Impressions

**Fig. 2:**
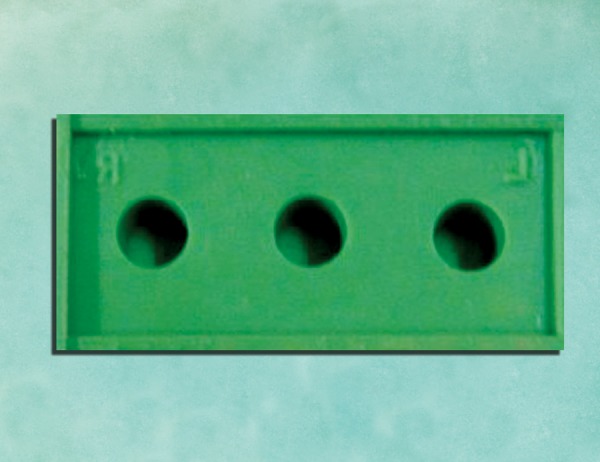
Single-step putty-wash impression technique

The impressions were categorized into four groups as follows:

*Group I: *One-step technique in which putty and wash impression materials were used simultaneously and the casts obtained from them were categorized as group I casts (Fig. 2).

**Figs 3A and B:**
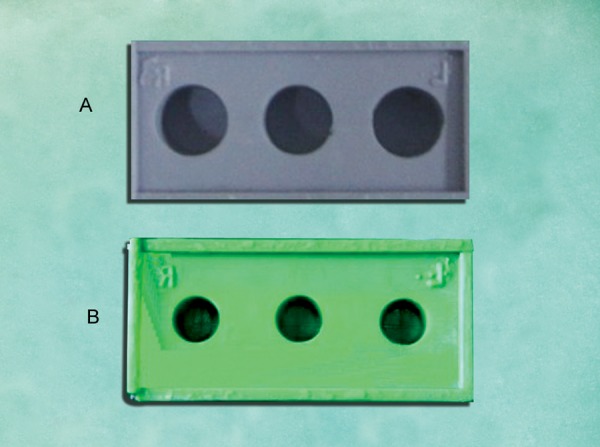
Putty-wash with copings as spacer

*Group II: *Two-step technique in which primary impression with putty was made with 1 mm thick metal copings placed over the abutments. The copings were removed to create a uniform 1 mm wash space. Wash impression material was syringed around the abutments and the primary putty impression was seated to get a complete two-step putty- wash impression. The casts obtained from them were categorized as group II casts (Fig. 3).

*Group III: *Two-step technique in which primary impression with putty was made with 2 mm thick metal copings placed over the abutments. The copings were removed to create a uniform 2 mm wash space. Wash impression material was syringed around the abutments and the primary putty impression was seated to get a complete two-step puttywash impression. The casts obtained from them were categorized as group III casts (Fig. 3).

**Fig. 4:**
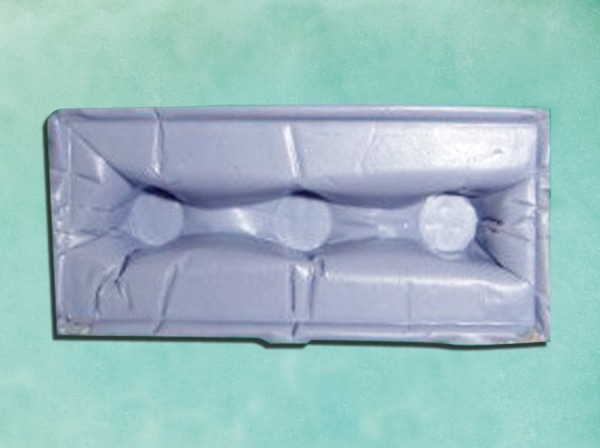
Putty-wash with polyethylene spacer

*Group IV: *Two-step technique in which a polyethylene spacer was used with putty impression and later the polyethylene spacer was removed to create a wash space. The wash impression material was syringed around the abutments and the putty impression was seated to get a complete two-step putty-wash impression. The casts obtained from them were categorized as group IV casts (Fig. 4).

### Measuring Procedure

**Fig. 5:**
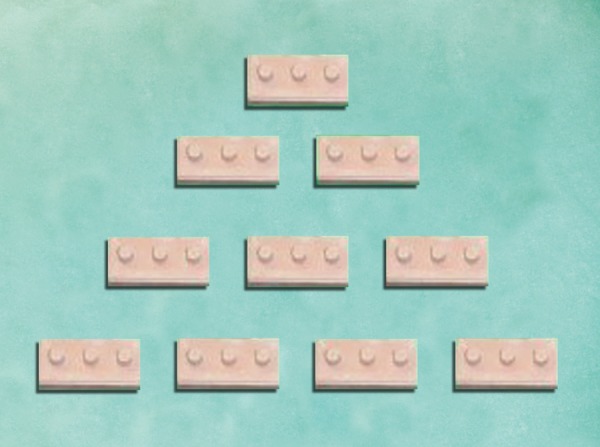
Putty-wash with copings as spacer

**Fig. 6:**
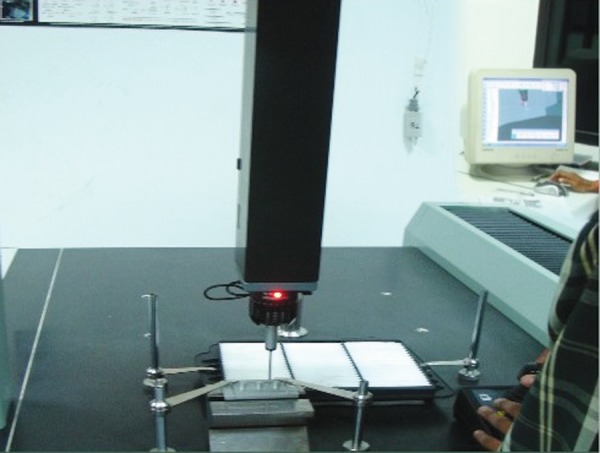
Measurement with coordinate measuring machine

The measurements of master model and stone casts (Fig. 5) were done using coordinate measurement machine (three-dimensional measurement machine) (Fig. 6) with accuracy up to 0.001 mm. It is mechanical system designed to move a measuring probe to locate reference points on the occlusal and horizontal platform. It consists of four components: The machine itself, measuring probe, the control or computing system and measuring software. The probe used can be either mechanical optical or a laser probe.

## OBSERVATION AND RESULTS

The difference between the mean of stone model (msm) and mean of master model (mmm) divided by mean of master model multiplied by 100 was expressed as percentage deviation from master model for each impression technique of each measurement location:

Percentage of deviation = (msm – mmm)/mmm × 100

**Fig. 7:**
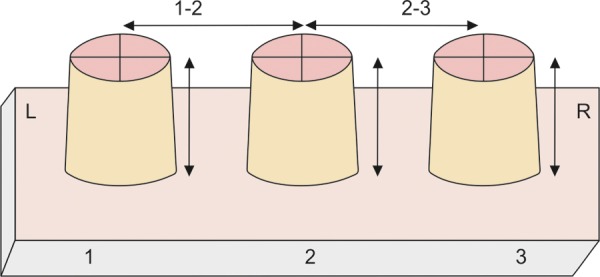
The various distances measured were intra-abutment (vertical) and interabutment (horizontal)

All the measurements obtained for all four groups were tabulated and statistically analyzed (Tables 1 to 4 and Fig. 7).

## DISCUSSION

**Graph 1:**
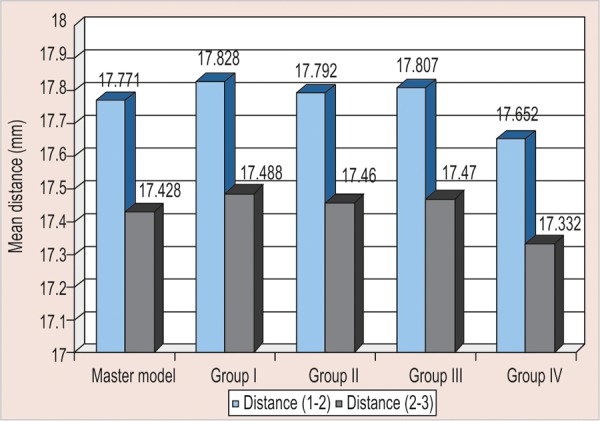
Mean of interabutment distances

**Graph 2:**
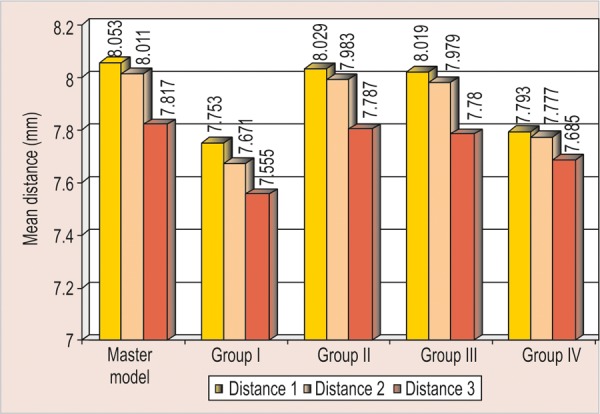
Mean of intra-abutment distances (1, 2 and 3)

**Graph 3:**
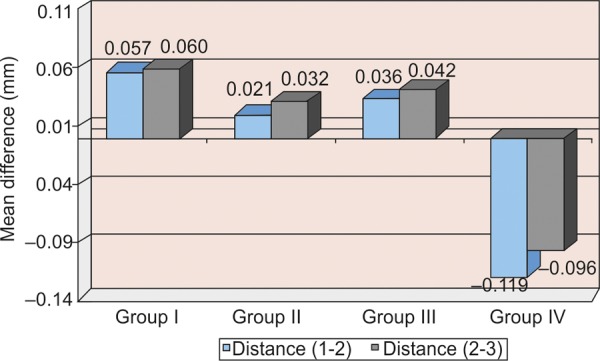
Mean difference of interabutment distances (1-2 and 2-3) between casts and master model

**Graph 4:**
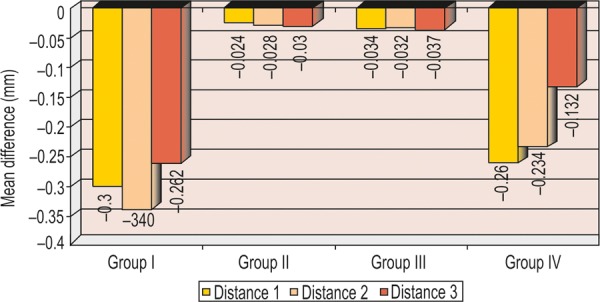
Mean difference of intra-abutment distances (1, 2 and 3) between casts and master model

- The aim of the present study was to compare the accuracy of stone casts obtained from different putty/wash impression techniques using various spacer thickness (Graphs 1 to 4).
- The critical factor that influences the accuracy of putty- wash impression techniques is the controlled wash bulk which is absent in one-step putty-wash impression technique and with polyethylene spacer was used.
- The above results showed that when stone casts and master model were compared, the vertical distance (intra- abutment) of the stone dies decreased, whereas horizontal distance (interabutment) increased.
- In the present study, the controlled wash space is essential for accuracy of putty-wash impressions. The controlled wash space was provided by uniform spacer thickness of 1 and 2 mm. The uncontrolled wash bulk was seen in one-step impression technique and two-step impression technique with polyethylene spacer.
- The results of present study do not agree with Hung et al and Idris et al. Hung et al and Idris et al investigated the importance of impression techniques and reported that impression accuracy is not technique dependent.
- Based on the observation of the present study, two-step putty-wash technique with 1 and 2 mm spacer thickness is more acceptable and viable alternative to obtain accurate impressions.
- The clinical implication of the present study is that to achieve a controlled wash bulk, temporary crowns can be used to create the desired wash space in the putty impression.
- Further investigation is needed to determine the exact amount and technique of achieving wash space that is essential for accuracy in using two-step putty/wash impression techniques in conjunction with polyvinyl siloxane impression materials. Study can also be undertaken for dimensional accuracies in long-span bridges.

**Table 1:** **Table 1: **Measurements of interabutment distances on the master model and stone casts for all four groups in mm

*S. no.*				*Master model*				*Group I*				*Group II*				*Group III*				*Group IV*			
				1-2		2-3		1-2		2-3		1-2		2-3		1-2		2-3		1-2		2-3	
1				17.771		17.428		17.826		17.49		17.788		17.456		17.807		17.473		17.648		17.336	
2								17.825		17.491		17.789		17.455		17.808		17.464		17.662		17.341	
3								17.828		17.484		17.79		17.464		17.806		17.476		17.645		17.334	
4								17.829		17.486		17.792		17.458		17.817		17.463		17.644		17.328	
5								17.83		17.489		17.791		17.465		17.816		17.472		17.653		17.325	
6								17.823		17.488		17.795		17.459		17.809		17.466		17.646		17.339	
7								17.827		17.49		17.798		17.462		17.794		17.471		17.663		17.324	
8								17.833		17.487		17.793		17.463		17.805		17.473		17.643		17.332	
9								17.824		17.489		17.797		17.457		17.802		17.466		17.665		17.326	
10								17.834		17.491		17.787		17.46		17.804		17.473		17.651		17.338	

**Table 2:** **Table 2: **Measurements of intra-abutment distances on the master model and stone casts for all the four groups in mm

*S. no.*				*Master model*						*Group I*						*Group II*						*Group III*						*Group IV*					
				*1*		2		3		*1*		2		3		*1*		2		3		*1*		2		3		*1*		2		3	
1				8.053		8.011		7.817		7.753		7.673		7.551		8.032		7.983		7.788		8.022		7.982		7.776		7.796		7.786		7.685	
2										7.758		7.676		7.55		8.03		7.986		7.785		8.024		7.983		7.78		7.793		7.782		7.689	
3										7.748		7.671		7.548		8.026		7.98		7.79		8.02		7.98		7.779		7.79		7.781		7.69	
4										7.751		7.68		7.56		8.029		7.985		7.783		8.019		7.979		7.783		7.786		7.775		7.695	
5										7.752		7.666		7.562		8.033		7.981		7.784		8.025		7.977		7.777		7.8		7.77		7.678	
6										7.756		7.668		7.555		8.027		7.984		7.786		8.017		7.98		7.781		7.806		7.771		7.676	
7										7.76		7.664		7.559		8.025		7.982		7.792		8.014		7.975		7.784		7.78		7.772		7.692	
8										7.752		7.669		7.558		8.031		7.979		7.787		8.018		7.971		7.771		7.799		7.77		7.677	
9										7.747		7.675		7.554		8.034		7.987		7.784		8.011		7.982		7.779		7.791		7.789		7.681	
10										7.755		7.672		7.553		8.024		7.983		7.789		8.019		7.984		7.786		7.789		7.776		7.683	

**Table 3:** **Table 3: **Mean values, standard deviation, deviation of interabutment distances from master model of all the groups

*Interabutment distance*		*Master model*				*Group 1*				*Group II*				*Group III*				*Group IV*		
		*1-2*		*2-3*		*1-2*		*2-3*		*1-2*		*2-3*		*1-2*		*2-3*		*1-2*		*2-3*
Mean (mm)		17.771		17.428		17.83		17.49		17.79		17.46		17.81		17.47		17.65		17.33
Standard deviation						0.004		0.002		0.004		0.0035		0.007		0.005		0.008		0.006
Deviation from master model (mm)						0.057		0.060		0.021		0.032		0.036		0.042		-0.119		-0.096
Deviation from master model (μm)						57		60		21		32		36		42	-119		-96	
Percent of deviation						0.321		0.344		0.118		0.184		0.203		0.241		-0.670		-0.551

**Table 4:** **Table 4: **Mean values, standard deviation, deviation of intra-abutment distances from master model of all the groups

*Intra-abutment distance*		*Master model*						*Group I*						*Group II*						*Group III*						*Group IV*					
		*1*		2		3		*1*		2		3		*1*		2		3		*1*		2		3		*1*		2		3	
Mean (mm)		8.053		8.011		7.817		7.753		7.671		7.555		8.029		7.983	7.87			8.019	7.8		7.78			7.793		7.777		7.685	
Standard deviation							-0.004		-0.005		-0.004		-0.004		-0.003		-0.003		-0.004		-0.004		-0.004		-0.083		-0.007		-0.007		
Deviation from master							-0.300		-0.340		-0.262		-0.024		-0.028		-0.030		-0.034		-0.037		-0.037		-0.260		-0.234		-0.132		
model (mm)																														
Deviation from master							-300		-340		-262		-24		-28		-30		-34		-37		-37		-260		-234		-132		
model (μm)																														
Percent of deviation							-3.725		-4.244		-3.352		-0.298		-0.35		-0.384		-0.422		-0.473		-0.473		-3.229		-2.921		-1.689		

## SUMMARY AND CONCLUSION

1. A two-step technique with uniform and controlled wash space is recommended for the fabrication of stone dies which will result in precise fitting of the restoration.
2. The two-step putty-wash technique with 1/2 mm spacer thickness produced casts within accepted clinical range. The one-step and two-step with polyethylene spacer produced the most uneven dimensional changes.
3. The clinical implication of this study will be to use temporary crowns to create controlled wash space.
